# Minimally Invasive Anatomical Segmentectomy versus Lobectomy in Stage IA Non-Small Cell Lung Cancer: A Systematic Review and Meta-Analysis †

**DOI:** 10.3390/cancers14246157

**Published:** 2022-12-14

**Authors:** Luca Bertolaccini, Elena Prisciandaro, Claudia Bardoni, Andrea Cara, Cristina Diotti, Lara Girelli, Lorenzo Spaggiari

**Affiliations:** 1 Department of Thoracic Surgery, IEO, European Institute of Oncology IRCCS, 20141 Milan, Italy; 2 Department of Oncology and Hemato-Oncology, University of Milan, 20141 Milan, Italy

**Keywords:** segmentectomy, lobectomy, lung cancer, robot-assisted thoracic surgery, video-assisted thoracic surgery, systematic review, meta-analysis

## Abstract

**Simple Summary:**

The detection rate of small peripheral lung nodules has risen dramatically due to the advances in lung cancer screening programs. Approximately 10% of these nodules are malignant non-small cell lung cancer (NSCLC). While current EVIDENCE suggests that lobectomy and segmentectomy have comparable outcomes for patients with stage IA NSCLC, certain studies have suggested that segmentectomy has a worse prognosis than lobectomy. Furthermore, the superiority of segmentectomy in retaining pulmonary function remains debatable. The Japanese randomized control trial JCOG0802/WJOG4607L was the first phase 3 trial to demonstrate the superiority of segmentectomy over lobectomy in terms of overall patient survival, indicating that segmentectomy should become the standard surgical treatment for early-stage patients. Our study aimed to conduct a systematic review and meta-analysis to establish whether minimally invasive anatomical segmentectomy and lobectomy had comparable perioperative and survival outcomes in early-stage NSCLC patients.

**Abstract:**

Objective. A systematic review and meta-analysis was performed to assess potential differences in perioperative outcomes and disease-free survival (DFS) and overall survival (OS) of patients with pathological stage IA non-small cell lung cancer (NSCLC) who underwent minimally invasive anatomical segmentectomy or lobectomy. Methods. This systematic review and meta-analysis followed the Preferred Reporting Items for Systematic Reviews and Meta-Analyses (PRISMA) guidelines. A systematic search of EMBASE (through Ovid), MEDLINE (via PubMed), and Cochrane CENTRAL was conducted. Two researchers independently reviewed each eligible study that included patients with stage IA NSCLC who underwent minimally invasive anatomical segmentectomy and lobectomy and compared perioperative and/or survival outcomes of patients. Results. A total of 887 publications were identified. Of these, 10 articles met our eligibility criteria. A significantly higher number of lymph nodes were harvested in lobectomies. The two groups did not significantly differ in postoperative complication rates, DFS, and OS. Patients who underwent segmentectomy had shorter postoperative hospital stays. Conclusions. Minimally invasive lobectomy and segmentectomy showed comparable short-term and long-term outcomes in stage IA NSCLC patients. Postoperative complication rates were similar. Minimally invasive lobectomies are associated with a higher number of harvested lymph nodes, although this did not affect the final staging or the survival outcomes.

## 1. Introduction

The detection rate of small peripheral lung nodules has risen dramatically due to the advances in lung cancer screening programs. Approximately 10% of these nodules are malignant non-small cell lung cancer (NSCLC) [1,2]. In 1995, the North American Lung Cancer Study Group revealed superior patient survival after lobectomy than after wedge resections, confirming lobectomy with radical lymph node dissection as the gold standard treatment for stage IA NSCLC [3]. The study did not differentiate between wedge resections and segmentectomies and included patients at various clinical stages [4]. While current evidence suggests that lobectomy and segmentectomy have comparable outcomes for patients with stage IA NSCLC, certain studies have suggested that segmentectomy has a worse prognosis than lobectomy. Furthermore, the superiority of segmentectomy in retaining pulmonary function remains debatable. The Japanese randomized control trial JCOG0802/WJOG4607L was the first phase 3 trial to demonstrate the superiority of segmentectomy over lobectomy in terms of overall patient survival, indicating that segmentectomy should become the standard surgical treatment for early-stage patients [5].

Nonetheless, segmentectomies differ in terms of operational difficulty. The anatomical challenges of segmentectomies, especially in the case of nonpalpable tumors (e.g., ground-glass opacities (GGO)), may hinder the achievement of negative resection margins and a radical hilar lymph node dissection. The most demanding aspect of segmentectomies is identifying the intersegmental plane. Segmentectomies can be classified as “easy” or “complex” procedures based on the number and shape of intersegmental planes [6]. Several challenges still exist regarding the extent of curative lung resection, and minimally invasive lobectomy and segmentectomy have been refined over the past decade [7]. In addition, current research suggests that pure GGO are amenable to segmentectomy, thus generating selection bias due to the inferior aggressiveness and more favorable oncological prognosis of GGO themselves [8].

This study aimed to conduct a systematic review and meta-analysis to establish whether minimally invasive anatomical segmentectomy and lobectomy had comparable perioperative and survival outcomes in early-stage NSCLC patients.

## 2. Material and Methods

**Figure 1:**
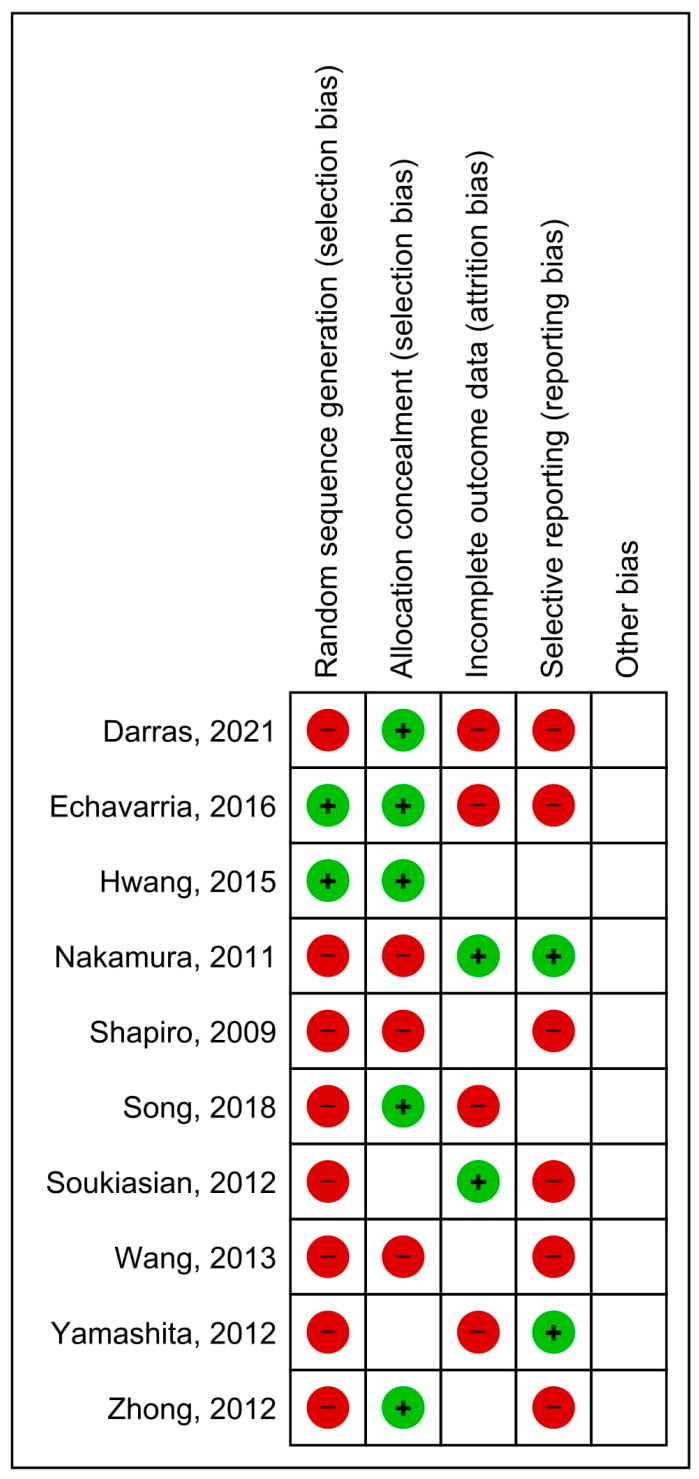
Risk of bias summary. Authors’ assessments of each risk of bias item for each study included. The green and red circles correspondingly represent a low and high risk of bias [8,12,13,14,15,16,17,18,19,20].

This systematic review and meta-analysis adhered to the Preferred Reporting Items for Systematic Reviews and Meta-Analyses (PRISMA) standards [9,10,11]. Combining free-text words, suitable MeSH headings, and limitations, a search technique was devised (time limit: January 1990 to March 2022; language: English). There was a systematic search of MEDLINE (via PubMed), EMBASE (through Ovid), and Cochrane CENTRAL. We included studies aimed at comparing minimally invasive anatomical segmentectomy and lobectomy for stage IA NSCLC in terms of perioperative and/or survival outcomes. The exclusion criteria included editorials, letters, case reports, expert opinions, propensity score-matching analyses, reviews, and meta-analyses. Our search results were imported into a reference management application. In instances of document duplication, the latest document was chosen. In order to select relevant articles for the meta-analysis, a thorough full-text review was conducted on potentially eligible papers, following an initial title/abstract screening. Each qualified study was independently reviewed by two researchers (EP and CB). We noted the following data: first author’s name, geographic region, publication year, study design, overall survival (OS), disease-free survival (DFS), number of harvested lymph nodes, postoperative complications, and length of postoperative hospital stay. Disagreements were resolved by discussion with a senior author (LB). This systematic review protocol was registered in the International Prospective Register of Systematic Reviews (PROSPERO, https://www.crd.york.ac.uk/PROSPERO/, accessed on 31 July 2022): CRD42016040153. A PRISMA checklist was added (Supplementary File S1). Figure 1 provides an overview of the risk of bias for each included study.

### Statistical Analysis

For dichotomous variables, the Mantel–Haenszel formula was used to produce a pooled effect estimate in the form of risk ratios and their related 95% confidence intervals (CI). The mean differences (MD) for continuous outcomes were aggregated, weighted by generic inverse variance, and evaluated using random effects modelling. Means and standard deviations were determined when continuous results were presented in some research as median, range, and interquartile range. Using the hazard ratio (HR) and standard error (SE), we analyzed survival statistics (OS and DFS) [21,22]. If HR data could not be obtained directly from the included studies, we extracted data from Kaplan–Meier curves and computed the data using the technique described in the scientific literature (10). A statistically significant global effect was defined as a *p*-value of 0.05 or a 95% confidence interval that did not cross the line indicating no effect. The Higgins *I*^2^ statistic was employed to measure heterogeneity. This statistic represents the fraction of total variation between studies that can be attributed to heterogeneity as opposed to random variation. The research indicates that a score of 25% indicates low heterogeneity, 50% indicates moderate heterogeneity, and 75% indicates significant heterogeneity (11). In the absence of significant heterogeneity, the fixed-effects model was applied to combine studies; otherwise, the random-effects model was used. Several possible sources of bias were evaluated, including allocation concealment, random sequence creation, blinding, inadequate outcome data, selective result reporting, and others. The instrument developed by the Cochrane Collaboration was used to assess the risk of bias for the primary outcome of included studies [23]. At the outcome level, the risk of bias due to incomplete outcome data was evaluated. At the study level, the risk of bias originating from sequence generation, blinding, allocation concealment, funding, or selective reporting was evaluated. Two independent reviewers (CB, EP) determined the possibility of bias, and disputes were resolved by discussion and consensus with a senior author (LB). Review Manager 5.4.1 (Nordic Cochrane Centre, Copenhagen, Denmark, https://training.cochrane.org/online-learning/core-software/revman/revman-5-download, accessed on 11 January 2022) and RStudio (R version 4.2.1, Funny-Looking Kid) were utilized for data analyses [24,25].

## 3. Results

**Figure 2:**
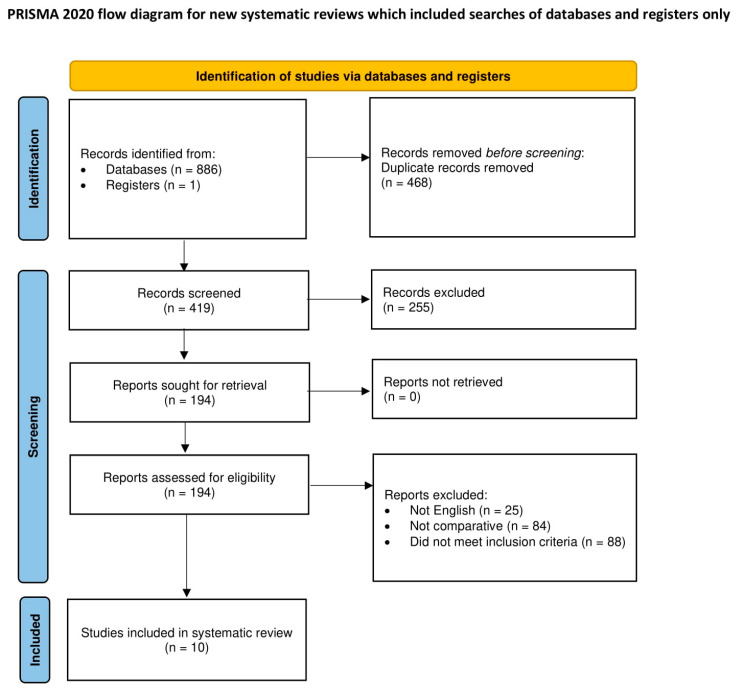
PRISMA 2020 flow schematic of the search approach used to discover pertinent comparative studies on minimally invasive segmentectomy versus lobectomy.

The manual search of reference lists and the electronic database search yielded a total of 887 publications. Among them, 10 retrospective studies met our eligibility criteria (Figure 2) for a total of 1953 patients: 550 underwent minimally invasive anatomical segmentectomy, and 1403 underwent minimally invasive lobectomy [8,12,13,14,15,16,17,18,19,20]. The characteristics of the included papers are presented in Table 1.

**Figure 3:**
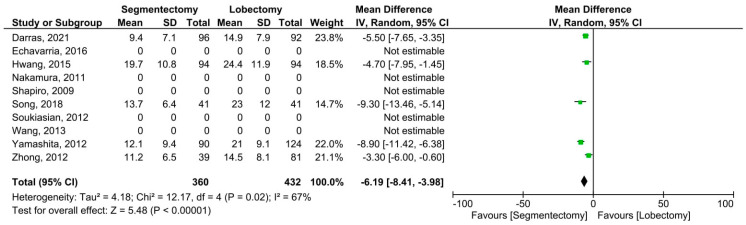
Forest plot for harvested lymph nodes of minimally invasive segmentectomy and lobectomy. CI = confidence interval; dF = degree of freedom; IV = inverse variance; SD = standard deviation [8,12,13,14,15,16,17,18,19,20].

**Figure 4:**
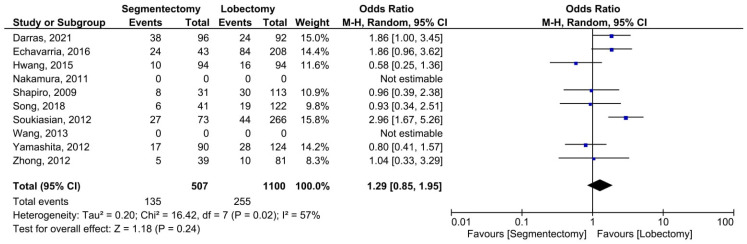
Forest plot for postoperative complications of the minimally invasive segmentectomy and lobectomy. CI = confidence interval; dF = degree of freedom; M–H = Mantel–Haenszel [8,12,13,14,15,16,17,18,19,20].

**Figure 5:**
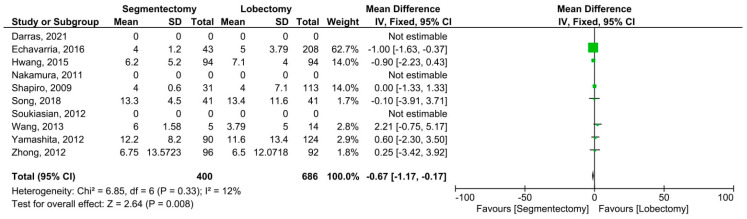
Forest plot for the minimally invasive segmentectomy and lobectomy postoperative hospital stay. CI = confidence interval; dF = degree of freedom; IV = inverse variance; SD = standard deviation [8,12,13,14,15,16,17,18,19,20].

Five articles reported data on harvested lymph nodes and involved 873 patients (360 received segmentectomies and 513 lobectomies). Significant heterogeneity among the studies was demonstrated (*I*^2^ = 67%, *p* = 0.02). A significantly higher number of lymph nodes was harvested in minimally invasive lobectomies (MD = −6.19, 95% CI: −8.41–−3.98, *p* < 0.00001, Figure 3). Eight articles including 1607 individuals published data on postoperative complications (507 minimally invasive segmentectomies and 1100 lobectomies). Significant heterogeneity was observed among the studies (*I*^2^ = 57%, *p* = 0.02). The incidence of postoperative complications did not change substantially between the two groups (OR = 1.29, 95% confidence interval: 0.85–1.95, *p* = 0.24, Figure 4). Seven publications with 1099 individuals published data on postoperative hospital stay (343 minimally invasive segmentectomies and 756 lobectomies). The studies found no significant heterogeneity (*I*^2^ = 12%, *p* = 0.33). Postoperative hospital stay was significantly shorter for patients who underwent minimally invasive segmentectomy (MD = −0.67, 95% CI: −1.17–−0.17, *p* = 0.008, Figure 5).

**Figure 6:**
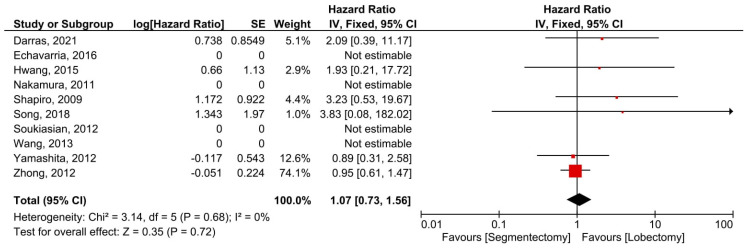
Forest plot for disease-free survival of the minimally invasive segmentectomy and lobectomy. CI = confidence interval; dF = degree of freedom; IV = inverse variance; SD = standard deviation [8,12,13,14,15,16,17,18,19,20].

**Figure 7:**
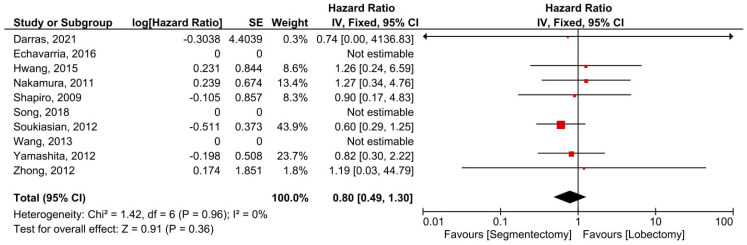
Forest plot for overall survival of the minimally invasive segmentectomy and lobectomy. CI = confidence interval; dF = degree of freedom; IV = inverse variance; SD = standard deviation [8,12,13,14,15,16,17,18,19,20].

Six manuscripts described data on DFS and concerned 1017 patients (391 minimally invasive segmentectomies and 626 lobectomies). There was no heterogeneity between trials (*I*^2^ = 0%, *p* = 0.68). The overall HR for DFS was 1.07 (95% confidence interval: 0.73–1.56). The DFS did not differ substantially between the two groups (*p* = 0.72, Figure 6). Seven studies presented OS data for 1520 individuals (461 minimally invasive segmentectomies and 1059 lobectomies) included in seven investigations. There was no heterogeneity between trials (*I*^2^ = 0%, *p* = 0.96). The combined HR for OS was 0.80 (95% confidence interval: 0.49 to 1.20). The OS did not differ substantially between the two groups (*p* = 0.36, Figure 7).

## 4. Discussion

Early diagnosis, tailored treatment, and long-term surveillance are crucial steps in lung cancer management. Over the past two decades, noteworthy progress has been achieved in detecting, diagnosing, and treating NSCLC. Low-dose computed tomography is effective for the early identification of lung cancer in high-risk individuals [1,2,26]. Surgical excision remains the mainstay treatment for NSCLC in its earliest stages [27]. Rigid adherence to contemporary oncologic principles and techniques is paramount, regardless of the surgical strategy. Radical tumor resection, safe surgical performance, and preservation of respiratory function must be considered.

However, the more suitable extent of resection for early-stage NSCLC is currently under discussion. The gold standard is a lobectomy, even if anatomical pulmonary segmentectomy has gained popularity during the past decade as a safe alternative for patients with impaired cardiopulmonary function. Screening programs for lung cancer have improved the detection of small nodules and GGO. As a result, the efficacy of lobectomy versus sublobar resection for early-stage NSCLC has been questioned [8]. Systematic reviews demonstrate that segmentectomy can obtain the same survival results as lobectomy in people with stage I NSCLC, with a paucity of studies on minimally invasive anatomical segmentectomy [28]. The variations in mortality outcomes, surgical complications, and the number of retrieved lymph nodes between segmentectomy and lobectomy have not been properly explored. Our study and meta-analysis included 10 articles that compared the perioperative and oncological outcomes of minimally invasive anatomical segmentectomy against lobectomy in patients with stage IA NSCLC. These retrospective studies were graded as being of poor to moderate quality. We discovered that the results for minimally invasive anatomical segmentectomy and lobectomy patients were comparable. There were no significant differences between the two groups in terms of OS and DFS and postoperative complications. However, individuals who underwent minimally invasive segmentectomy had considerably shorter postoperative hospital stays and fewer lymph nodes collected.

The JCOG0802/WJOG4607L study found that while surgical complications and median postoperative chest tube-dwelling time did not differ between lobectomy and segmentectomy, and patients who underwent segmentectomy were more likely to experience prolonged air leakage. The main objective of segmentectomies is to preserve pulmonary function, and the resection of two or five subsegments is associated with segmentectomy’s advantage of keeping higher pulmonary function than lobectomy. The JCOG0802/WJOG4607L reported that pulmonary function was more frequently preserved following segmentectomy [5]. While freedom from recurrence was comparable between lobectomy and segmentectomy, local recurrence at the surgical margin or within the preserved lobe is an inherent danger of segmentectomy [29].

The majority of trials indicating the superiority of lobectomy were not totally randomized and did not take into consideration other variables that could affect survival (4). Significantly fewer lymph nodes were removed with minimally invasive anatomical segmentectomy than during lobectomy. This may be owing to variances in the quantity of the inter-segmental and intra-segmental lymph nodes extracted, as well as the fact that lymph node sampling, as opposed to lymph node dissection, is typically performed during minimally invasive anatomical segmentectomy. However, in stage IA (pN0) patients, the number of harvested lymph nodes is not helpful for comparing lobectomies and segmentectomies since the DFS does not differ between the two techniques. However, three questions should be considered when analyzing clinical evidence: How reliable are the findings? Are the outcomes merely coincidental? Are the outcomes relevant to the patient? A typical issue is misinterpreting statistical significance as clinical importance. The clinical significance of study results is determined by their relevance to current clinical practice, with the degree of the treatment effect being one of the most important factors of treatment decisions. The clinical relevance of a change should be determined by its magnitude, its effect on patients’ lives, the length of its effects, consumer acceptance, cost-effectiveness, and ease of implementation. There are defined, generally accepted values for determining statistical significance, but none for judging clinical significance. Frequently, the clinician’s opinion determines whether or not a result is clinically meaningful. Even if statistically significant, the reported difference of a single lymph node across nodal locations is clinically inconsequential. It is essential to remember that the clinical significance of study results should be established by assessing the actual treatment impact (with confidence intervals) and not only based on *p*-values and statistical significance. If the outcome of interest does not get a stated minimum clinically meaningful difference, researchers should be mindful of interpretation outcomes as significant solely based on a *p*-value without considering the clinical significance of the data [30].

### Limitations

This systematic review and meta-analysis has a number of limitations. There was a substantial possibility of selection and reporting bias in each of the included retrospective, non-randomized studies. The high variation observed between studies in terms of postoperative complications, clinical stage, and the number of lymph nodes harvested may compromise the validity of the conclusions. Due to the existence of comorbidities, some patients who underwent minimally invasive segmentectomy were declared ineligible for lobectomy, leading in a selection bias that may have influenced these findings.

## 5. Conclusions

This systematic review and meta-analysis showed that minimally invasive lobectomy and anatomical segmentectomy have comparable short-term and long-term outcomes for stage IA NSCLC. In particular, OS and DFS did not significantly differ between the techniques, and postoperative complication rates and length of hospital stay were similar. Minimally invasive lobectomies were combined with a higher number of harvested lymph nodes, possibly owing to a deeper dissection in fissures. Future research should demonstrate theories using well-designed randomized controlled trials of minimally invasive lobectomies and anatomical segmentectomies (stratified for complexity) to investigate the effects on cost-effectiveness and outcome differences.

## Figures and Tables

**Table 1:** A summary of the studies that comprised the systematic review and meta-analysis. RATS = Robot-Assisted Thoracic Surgery; VATS = Video-Assisted Thoracic Surgery.

Authors	Year	Reference	Nation	Study Type	Surgical Approach	Minimally Invasives Segmentectomies	Minimally Invasive Lobectomies
Darras et al.	2021	[8]	Switzerland	Retrospective	VATS	96	92
Echavarria et al.	2016	[12]	USA	Retrospective	RATS	43	208
Hwang et al.	2015	[13]	Korea	Retrospective	VATS	94	94
Nakamura et al.	2011	[14]	Japan	Retrospective	VATS	38	289
Shapiro et al.	2009	[15]	USA	Retrospective	VATS	31	113
Song et al.	2018	[16]	Japan	Retrospective	VATS	41	122
Soukiasian et al.	2012	[17]	Japan	Retrospective	VATS	73	266
Wang et al.	2013	[18]	Japan	Retrospective	VATS	5	14
Yamashita et al.	2012	[19]	China	Retrospective	VATS	90	124
Zhong et al.	2012	[20]	China	Retrospective	VATS	39	81

## Supplementary Materials

The following are available online at www.mdpi.com/article/10.3390/cancers14246157/s1, Supplementary File S1: PRISMA Checklist.
