# Supplementary material for: Minimally Invasive Anatomical Segmentectomy versus Lobectomy in Stage IA Non-Small Cell Lung Cancer: A Systematic Review and Meta-Analysis
Source: Cancers (Basel). 2022 Dec 14;14(24):6157. doi: 10.3390/cancers14246157 (PMC9777177; doi:10.3390/cancers14246157)
Supplement: Supplementary file 1 [file cancers-14-06157-s001.zip › cancers-2070471-supplementary.pdf]

cancers

MDPI

Systematic Review

Minimally Invasive Anatomical Segmentectomy versus Lobectomy in Stage IA Non-Small Cell Lung Cancer: A Systematic Review and Meta-Analysis †

Luca Bertolaccini [1,*], Elena Prisciandaro [1], Claudia Bardoni [1], Andrea Cara [1], Cristina Diotti [1], Lara Girelli [1] and Lorenzo Spaggiari [1,2]

Supplementary File S1: PRISMA Checklist

exp LUNG NEOPLASMS/SU [SU=surgery]

(segmentectom* OR "limit* resect*" OR sublobar).ti,ab

(intention* OR compromis*).ti,ab

[exp LUNG NEOPLASMS/SU [SU=surgery]] AND [(segmentectom* OR "limit* resect*" OR sublobar).ti,ab] AND [(intention* OR compromis*).ti,ab]

(lung OR pulmo*).ti,ab

[(segmentectom* OR "limit* resect*" OR sublobar).ti,ab] AND [(intention* OR compromis*).ti,ab] AND [(lung OR pulmo*).ti,ab]

(VATS OR RATS OR “minim* invas*).ti,ab

[(segmentectom* OR "limit* resect*" OR sublobar).ti,ab] AND [(intention* OR compromis*).ti,ab] AND [(lung OR pulmo*).ti,ab] AND [(VATS OR RATS OR “minim* invas*).ti,ab]

[Limit to: (Document Status In Data Review or In Process)]
